# Computer Simulations of End-Tapering Anchorages of EBR FRP-Strengthened Prestressed Concrete Slabs at Service Conditions

**DOI:** 10.3390/ma16020851

**Published:** 2023-01-15

**Authors:** Chirawat Wattanapanich, Thanongsak Imjai, Reyes Garcia, Nur Liza Rahim, Mohd Mustafa Al Bakri Abdullah, Andrei Victor Sandu, Petrica Vizureanu, Petre Daniel Matasaru, Blessen Skariah Thomas

**Affiliations:** 1 School of Engineering and Technology, Walailak University, Nakhon Si Thammarat 80161, Thailand; 2 Civil Engineering Stream, School of Engineering, The University of Warwick, Coventry CV4 7AL, UK; 3 Faculty of Chemical Engineering Technology, Universiti Malaysia Perlis, Arau 02600, Malaysia; 4 Faculty of Material Science and Engineering, Gheorghe Asachi Technical University of Iasi, 41 D. Mangeron St., 700050 Iasi, Romania; 5 Romanian Inventors Forum, Str. Sf. P. Movila 3, 700089 Iasi, Romania; 6 National Institute for Research and Development for Environmental Protection INCDPM, 294 Splaiul Independentei, 060031 Bucharest, Romania; 7 Technical Sciences Academy of Romania, Dacia Blvd 26, 030167 Bucharest, Romania; 8 Faculty of Electronics, Telecommunications and Information Technology, Gheorghe Asachi Technical University of Lasi, Carol I 11A, 700506 Iasi, Romania; 9 National Institute of Technology Calicut, Calicut 673601, India

**Keywords:** FEA, end effect, EBR, plate bonding, tapering technique, prestressed concrete slab

## Abstract

This article examines numerically the behavior of prestressed reinforced concrete slabs strengthened with externally bonded reinforcement (EBR) consisting of fiber-reinforced polymer (FRP) sheets. The non-linear finite element (FE) program Abaqus^®^ is used to model EBR FRP-strengthened prestressed concrete slabs tested previously in four-point bending. After the calibration of the computational models, a parametric study is then conducted to assess the influence of the FRP axial stiffness (thickness and modulus of elasticity) on the interfacial normal and shear stresses. The numerical analysis results show that increasing the thickness or the elastic modulus of the FRP strengthening affects the efficiency of the FRP bonding and makes it susceptible to earlier debonding failures. A tapering technique is proposed in wet lay-up applications since multiple FRP layers are often required. It is shown that by gradually decreasing the thickness of the FRP strengthening, the concentration of stress along the plate end can be reduced, and thus, the overall strengthening performance is maximized. The tapering is successful in reducing the bond stress concentrations by up to 15%, which can be sufficient to prevent concrete rip-off and peel-off debonding failure modes. This article contributes towards a better understanding of the debonding phenomena in FRP-strengthened elements in flexure and towards the development of more efficient computational tools to analyze such structures.

## 1. Introduction

The need to increase service loads, changes or updates in design codes, design errors or aging make the strengthening of reinforced concrete elements necessary. Due to several advantages over more traditional techniques, externally bonded reinforcement (EBR) in the form of fiber-reinforced polymers (FRP) has gained a special interest in strengthening applications of reinforced concrete (RC) structures [1,2,3,4]. The most common technique to strengthen reinforced concrete elements in flexure consists of the simple bonding of FRP plates or fabrics on the elements’ soffit. The two basic techniques for FRP strengthening normally used in practical applications are (1) prefabricated systems by means of cold cured adhesive bonding, or (2) wet lay-up systems. The application depends on strengthening needs and the type of structure. In the case of FRP strips and laminates (prefab type), the adhesive ensures bonding, and therefore, a high viscosity thixotropic adhesive is applied. In the case of FRP sheets or fabrics (wet lay-up application), a resin ensures both bonding and impregnation of the sheets, and therefore, a low viscosity resin is often required. Since the tensile strength of FRP materials is several times higher than that of more traditional strengthening solutions (e.g., steel plates), the tensile strength of EBR FRP systems is much higher than its bonding strength to the concrete face. As a result, FRP debonding between concrete and EBR can become dominant in flexural elements strengthened with EBR FRP systems.

**Figure 1:**
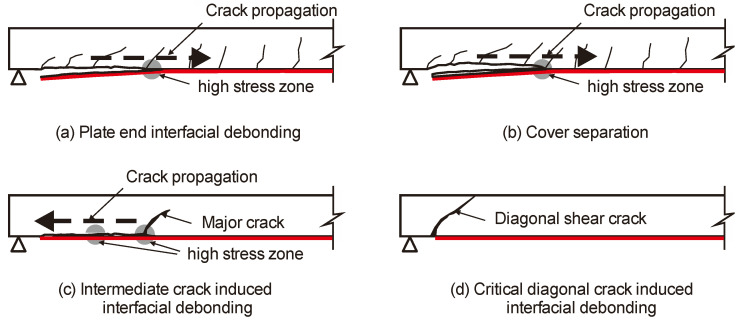
Typical EBR FRP debonding failure modes of RC slabs (adapted from [7]). (**a**) Plate end interfacial debonding; (**b**) Cover separation; (**c**) Intermediate crack induced interfacial debonding; (**d**) Critical diagonal crack induced interfacial debonding.

Previous research indicates that EBR FRP strengthening can increase the flexural capacity of elements in both service and ultimate conditions [5,6,7,8]. However, reinforced concrete elements strengthened with EBR FRP can also suffer from premature and brittle debonding of the FRP plate or sheets [6,7,8,9,10]. Typical EBR FRP debonding failure modes of RC slabs can include plate end interfacial debonding (Figure 1a), concrete cover separation (Figure 1b), intermediate crack-induced interfacial debonding (Figure 1c) or shear failure due to diagonal cracking (Figure 1d). An FRP debonding failure can prevent the strengthened element from reaching its theoretical ultimate capacity, while also reducing its ductility. Therefore, bond failures such as those shown in Figure 1a–d must be prevented. This is necessary because the design procedure for FRP-strengthened flexural elements assumes that the design moment after strengthening results from a full composite action between the EBR FRP system and the concrete element. Indeed, in a real design, designers only have to verify that bond failures (due to shear cracks, end anchorage or along the FRP) do not occur.

**Figure 2:**
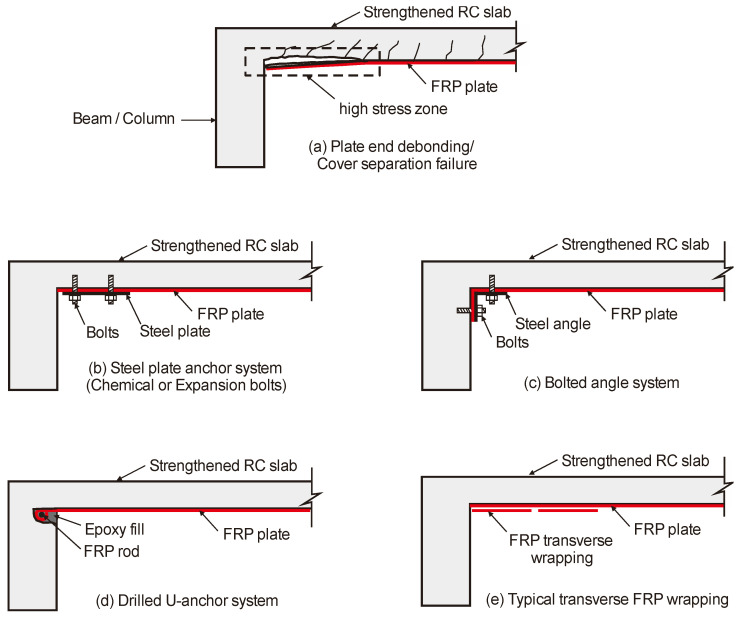
Schematic anchorage systems of EBR of RC slabs using FRP plate. (**a**) Plate end debonding/Cover separation failure; (**b**) Steel plate anchor system (Chemical or Expansion bolts); (**c**) Bolted angle system; (**d**) Drilled U-anchor system; (**e**) Typical transverse FRP wrapping.

In order to prevent debonding failures at the end anchorage of a plate or sheet of an EBR FRP-strengthened slab (Figure 2a), several solutions are used in practical applications [9]. For instance, steel plates and bolts (Figure 2b) or bolted steel angle systems (Figure 2c) were proposed. However, this implies that drilling (or fiber unknitting) is necessary to accommodate the bolts and anchor them into the slab or walls. Corrosion issues may also affect the plates or bolts, thus compromising the effectiveness of the FRP strengthening system. U-shaped anchorage systems were also used (Figure 2d). In this solution, the FRP sheets are extended beyond the edge of the slab into the support walls. The FRP sheets are then folded and anchored with a transverse bar or rod glued with epoxy resin. Another typical solution is the bonding of transverse FRP sheets to the slab soffit, placed normal to the (main) longitudinal direction of the flexural EBR FRP plate or sheets. The latter solution is by far the simplest to prevent end anchorage debonding failures, but it is also prone to experience premature debonding.

Several models/guidelines exist to predict the bond behavior of FRP-concrete systems, but they are still unable to give accurate predictions [6,7]. This inaccuracy can be attributed to a lack of understanding of the development of bond stress along the FRP–concrete interface, especially at the end plate section where high interfacial stress mobilizes and weakens the EBR FRP system [6]. Recent research [10] has found that, while numerous studies have examined the debonding of EBR FRP in flexural elements [11,12,13,14,15,16,17,18], much less research has focused on modeling numerically the behavior of such elements [19,20,21,22,23,24,25]. Moreover, parametric studies based on finite element analysis are particularly scarce and very much needed [10], as performing computer simulations is much more cost-effective than performing actual experiments.

Past studies have adopted different approaches to simulate debonding phenomena. For example, smeared and discrete approaches can be used to simulate cracking and debonding in plain and FRP-strengthened concrete structures [26,27]. Other numerical techniques to model the interaction between concrete and FRP systems assume a full composite action at the FRP–concrete interface so that no relative movement occurs between them [8]. Using this type of contact modeling, a full composite action develops and debonding is prevented, which has proven appropriate to model EBR FRP in reinforced concrete elements up to the service load level [5,6,7,8]. Despite the above advancements in computational approaches, the investigation of the modeling capabilities built in current finite element (FE) software was recently identified as a research need in EBR FRP-strengthened structures [11].

This article examines numerically the end-plate behavior of prestressed concrete slabs strengthened in flexure with EBR FRP sheets. This is achieved by using previous data from four-point bending tests on EBR FRP-strengthened prestressed concrete slabs. Non-linear FE analyses was first performed to calibrate computational models of the strengthened slabs. Subsequently, a parametric study was conducted to assess the influence of the FRP axial stiffness (i.e., thickness and modulus of elasticity) on the normal and shear interfacial stresses. The article also provides some recommendations to enhance the capacity of FRP bonded to prestressed concrete slabs, as well as to improve the effectiveness of FRP plate bonding techniques. This article contributes towards a better understanding of the debonding phenomena in FRP-strengthened elements in flexure and towards the development of more efficient computational tools to analyze such structures. 

## 2. Experimental Investigation 

**Figure 3:**
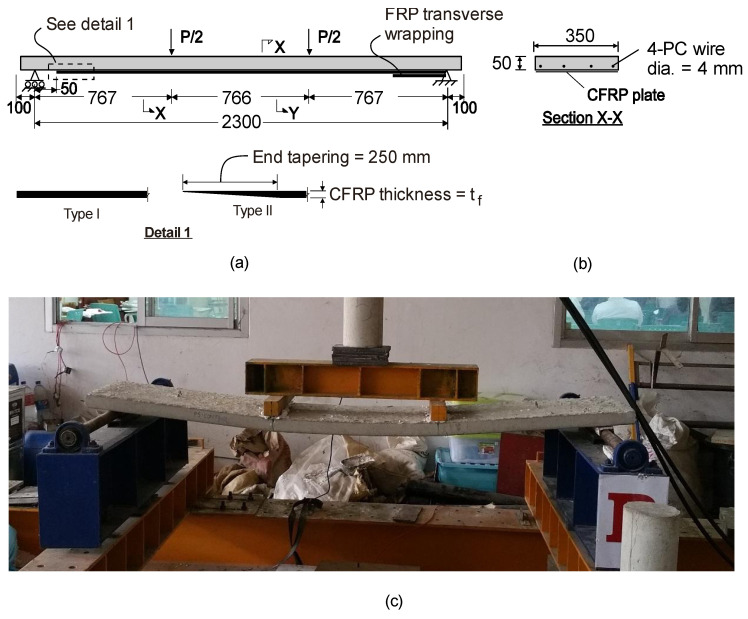
(**a**) Elevation and (**b**) cross section of slabs tested in four-point bending and (**c**) view of slab during test.

Five prestressed concrete slabs tested previously by the authors [8] were taken as case studies for calibrating FE models. Prestressed slabs are commonly used as floor systems in houses and small buildings in Southeast Asia, and, in many cases, such slabs need to be strengthened due to the change in use of the structure. The slabs had an effective span length of 3300 mm (Figure 3a) and a cross-section of 50 × 350 mm^2^ (Figure 3b). Four *f*4 mm prestressed tendons (*f_sy_* = 1860 MPa, *E_s_* = 205 GPa) were used as flexural bottom reinforcement. Such tendons complied with the ASTM A421 [28] specifications. The slabs were subjected to four-point bending (Figure 3c), with a shear span to effective depth ratio equal to 4.5. 

**Figure 4:**
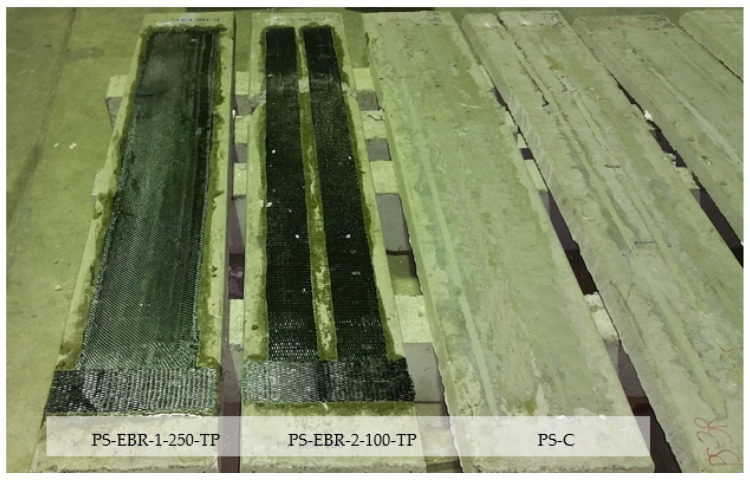
Application of EBR CFRP sheets at the slab’s soffit (PS-EBR-2-100).

One layer of carbon FRP (CFRP) strip with single or double layouts, having a thickness *t_f_*, of 1.4 mm, was used to strengthen the slabs’ soffit. The FRP sheets were bonded with the slabs in an upside-down position, as shown in Figure 4, that shows the following specimens:

- PS-EBR-1-250-TP: EBR CFRP-strengthened slab, strip width 250 mm, tapered at one end anchorage (tapering length = 250 mm).
- PS-EBR-2-100-TP: EBR CFRP-strengthened slab, strip width 100 mm each, tapered at one end anchorage (tapering length = 250 mm).
- PS-EBR-1-250 and PS-EBR-2-100: same as above but without tapering.
- PS-C: unstrengthened control slab.

The properties of the CFRP were modulus of elasticity *E_f_* = 200 GPa, Poisson ratio = 0.29, ultimate strength *f_fu_* = 2590 MPa and rupture strain ε*_fu_* = 0.015, as given by the manufacturer. The FRP was bonded to the slabs’ soffit using a two-part epoxy adhesive bonding agent. The properties of the epoxy resin reported by the producer were modulus of elasticity *E_m_* = 5 GPa, tensile strength *f_m_* = 20 MPa and Poisson ratio = 0.35. The slabs were cast using C30 concrete with a mean compressive strength of *f_c_* = 30.8 MPa, tensile splitting strength *f_cr_* = 3.6 MPa and modulus of elasticity *E_c_* = 22 GPa, which were calculated according to *fib* Model Code [29].

Table 1 summarizes the material parameters used in the FE modeling. As shown in Figure 4, the FRP sheets were anchored at one end with transverse CFRP sheets so as to promote FRP debonding on the opposite end anchorage of the slabs. 

**Figure 5:**
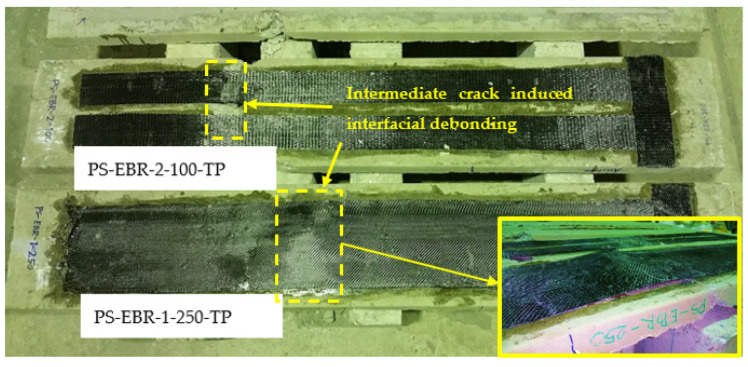
Intermediate crack-induced interfacial debonding failure of specimens with end-tapering.

**Figure 6:**
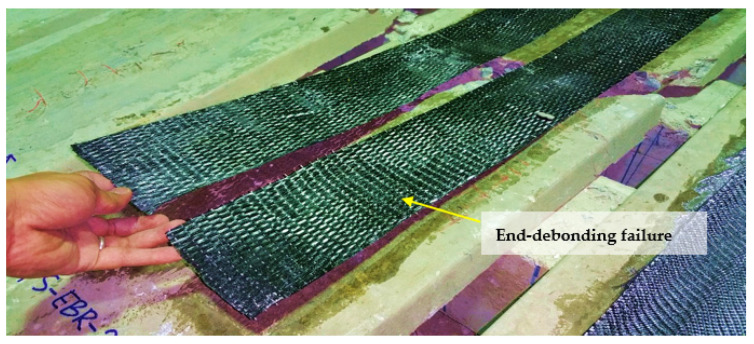
End-debonding failure of specimen without tapering (PS-EBR-2-100).

The five slabs were subjected to monotonic load until failure. The mid-span deflection behavior of the slabs was monitored using vertical displacement transducers located at the soffit. As expected, the ultimate failure of the slabs was controlled by debonding of the CFRP sheets at the free end anchorage for all of the strengthened specimens. Figure 5 shows the intermediate crack-induced interfacial debonding failure of specimens with tapering-end CFRP sheets (PS-EBR-2-100-TP and PS-EBR-1-250-TP). For the specimen without tapering (PS-EBR-2-100), end-debonding failure of CFRP was observed at the left side of the specimen, as shown in Figure 6.

## 3. Numerical Investigations

### 3.1. Modeling Assumptions

**Figure 7:**
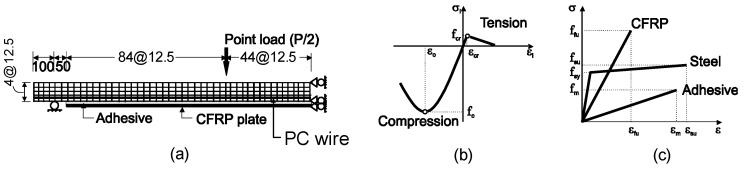
(**a**) FE mesh and geometry, (**b**) concrete constitutive model and (**c**) reinforcement (CFRP/steel/adhesive).

The prestressed concrete slabs were modeled in Abaqus^®^ FE software [30]. The concrete, adhesive layer (two-part epoxy resin) and CFRP sheets were modeled using two-dimensional solid biquadratic elements (CPS8) with eight-nodes (two degrees of freedom per node), as shown in Figure 7a. It should be noted that CPS4 or CPS4R elements consume less computation cost, but they were deemed unsuitable as they can cause shear stress deformation instead of bending deformation [30]. Figure 7b,c show the constitutive models of concrete and steel tendons used in Abaqus^®^. The internal reinforcement was modeled using 1D elements and is embedded in the concrete matrix, (*E*_s_ = 206 GPa and Poisson’s ratio = 0.3 (see Figure 7c). The prestressed tendons were ignored in the modeling. Instead, an equivalent prestressing force was applied as surface loads at both sides of the concrete elements, as reported in the previous studies by Okumus et al. [31]. Bond slip and dowel action were not explicitly considered in this study, primarily because the numerical simulations focused on the behavior of the slabs at the serviceability level where no major cracks or local slip were not evident in the experiments [4]. The concrete and CFRP sheets were assembled by a tie type of constraint so that no relative movement between them occurred. Using this type of contact modeling, a full composite action developed. As a result, the modeling of links and transverse CFRP sheets could be omitted. The analysis was performed by incremental loading, with integration in each increment. Since considerable nonlinearity was expected (including the possibility of instability as the concrete cracks or failure occurred at the FRP–concrete bond interface), the load magnitudes were covered by a single scalar parameter. The modified Riks algorithm with automatic increments was used. This method uses the “arc length” along the static equilibrium path in load-displacement space. This method in general worked well and provided a conservative solution for similar problems [30]. The FE method has proven effective at predicting with reasonable accuracy experimental results in previous studies [23,31,32].

### 3.2. Concrete Constitutive Model

Concrete was assumed to behave nonlinearly in both compression and tension, as shown in Figure 7b. The constitutive curve for concrete in compression is used as input parameters to the FE code in a form of stress–strain ($\sigma_{c}-\varepsilon_{c})$ series of values, as summarized in Table 2. By using the *fib* Model Code 2010 [29] approach, the FE analysis adopted the following stress–strain ($\sigma_{c}-\varepsilon_{c})\;$constitutive model for concrete in compression:

(1) $$\sigma_{c}=-f_{c}^{\prime }\frac{\left(\frac{E_{ci}}{E_{c1}}\right)\left(\frac{\varepsilon_{ci}}{\varepsilon_{c1}}\right)-{\left(\frac{\varepsilon_{ci}}{\varepsilon_{c1}}\right)}^{2}}{1+\left(\frac{E_{\mathrm{ci}}}{E_{c1}\;}-2\;\right)\left(\frac{\varepsilon_{ci}}{\varepsilon_{c1}}\right)}\;$$

where $E_{1}=\left|\frac{{f^{\prime }}_{c}}{\varepsilon_{1}}\right|$, $E_{ci}=E_{cm}{\left(\frac{f_{c}^{\prime }}{f_{cm0}}\right)}^{\frac{1}{3}}$; $\varepsilon {\;}_{1}=-0.0022\;$and $f_{cm0}=10\;$Mpa.

In Equation (1), $\varepsilon_{c}$ is the strain at extreme fiber that depends on the compressive strength of concrete$\;f_{c}^{\prime }$. The values of $E_{cm}$ adopted in this study correspond to the secant elastic modulus of concrete, which was derived according to *fib* Model Code 2010 [29].

## 4. Predictions of Slab Deflections 

**Figure 8:**
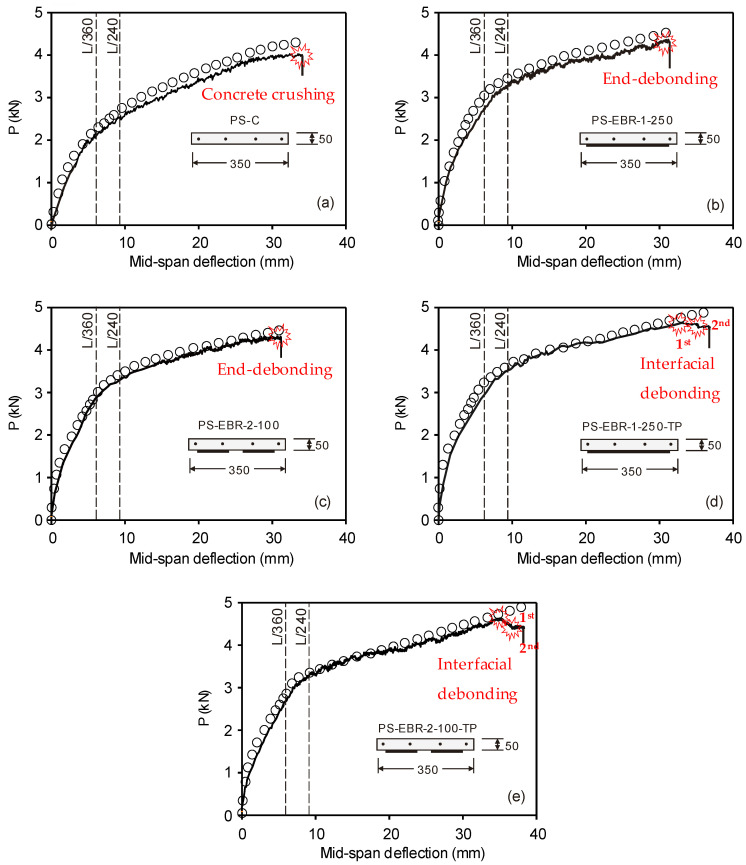
Comparison of experimental load-deflection and numerical predictions. (**a**) PS-C; (**b**) PS-EBR-1-250; (**c**) PS-EBR-2-100; (**d**) PS-EBR-1-250-TP; (**e**) PS-EBR-2-100-TP.

Figure 8a–e compares experimental load-deflection with the numerical predictions given by the FE models. For the control specimen (PS-C), the specimen failed by concrete crushing at the ultimate load of 3.98 kN and deflection of 32.8 mm. When considering the deflection limit of L/360 = 2300/360 = 6.38 mm, this occurs at a load level of 2.15 kN. The load level increases to 2.56 kN at L/240, as well as the deflection limit for a strengthened prestressed slab. The design live load of this slab is 1.5 kN for typical houses, and therefore, the test result evidenced that the design of the control PS-C was satisfactory. In the case of the strengthened slabs without tapering PS-EBR-1-250 and PS-EBR-2-100 (Figure 8b,c), it was found that the end-debonding failure (i.e., Figure 6) occurred. This occurred at the ultimate loads of 4.35 kN and 4.20 kN, respectively, as shown in Figure 8b,c. When considering the load level at deflection limit of L/360, it was found that the load level of PS-EBR-1-250 and PS-EBR-2-100 increased to 2.71 kN and 2.82 kN, respectively, which were 126% and 131% higher compared to the control PS-C.

For CFRP-strengthened specimens with tapering CFRP sheets at the end anchorage (PS-EBR-1-250-TP and PS-EBR-2-100-TP), it was found that plate end bonding failure was well prevented, and instead, multiple intermediate cracks induced interfacial debonding failures (i.e., Figure 5). This is reflected in the load-mid-span deflection curves in Figure 8d,e as sudden drops in the load towards the end of the tests. Generally, the first interfacial debonding failures occurred at the ultimate load, i.e., at 4.50 kN and 4.52 kN. This was followed by the second interfacial debonding failures at 4.23 kN and 4.12 kN for specimens PS-EBR-1-250-TP and PS-EBR-2-100-TP, respectively. When considering the load level at deflection limit of L/360 for PS-EBR-1-250-TP and PS-EBR-2-100-TP, it was found that the load level increased to 3.91 kN and 3.78 kN, respectively, which were 155% and 150% more than the equivalent loads of PS-C. From the results in Figure 8, it is concluded that all EBR CFRP-strengthened slabs improved both service and ultimate loads (by up to 15%) and stiffness (by up to 7%) over the control PS-C slab. It is also evident that the end tapering technique with CFRP sheets improved the ultimate load by up to 5% compared to their identical specimens, thus, also preventing end debonding failure by allowing a more gradual interfacial debonding failure.

The results in Figure 8a–e also show that the higher the load, the stiffer the response is obtained from the FE results compared to the experimental results (e.g., defection at failure from FE model is 13.3 mm vs. 17.1 mm from PS-C). Nevertheless, the FE models predict reasonably well the load and corresponding deflection up to 2.25 kN, which is considered sufficient to represent the point of service condition (i.e., a deflection = L/360). This is also consistent with the findings reported in previous studies [6,7,8]. At higher load levels, the deflections obtained from FE models are lower than the experimental values due to several cracks being developed after the service load level is reached. For CFRP-strengthened slabs, the FE models predict better the results compared to unstrengthened specimens up to a load level of approximately 75% of the ultimate load level. Moreover, the FE models predicts 20–25% lower deflections at ultimate load. It is also noted that the numerical prediction in this study did not capture the failure due to end-debonding or interfacial debonding (i.e., Figure 1), since perfect bonding between CFRP and concrete surface was assumed in the analysis, and thus, no separation was allowed to occur in the FE analysis. It is also interesting that numerical predictions agreed well with experimental results after service load level. This can be attributed to the fact that major cracks are prevented by the CFRP sheets bonded at the slabs’ soffit.

## 5. Parametric Studies

**Figure 9:**
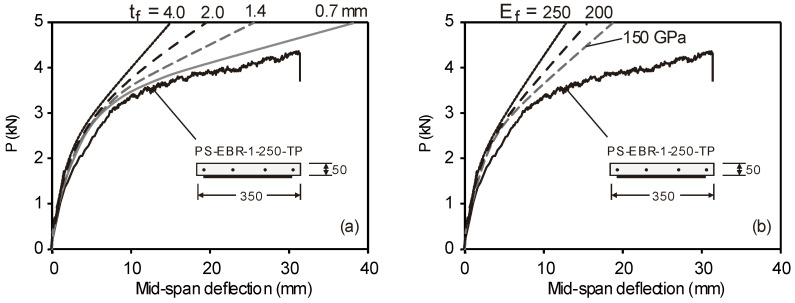
Effects of (**a**) thickness of CFRP, and (**b**) and modulus of elasticity of CFRP.

A parametric study is performed on slab PS-EBR-1-250-TP to investigate the stress in the flexural CFRP sheets and find solutions to make the strengthening more effective by varying the axial CFRP stiffness (thickness and modulus of elasticity). Figure 9a shows the load-deflection curves of strengthened slabs with different fiber thicknesses (*t_f_* = 0.7, 1.4, 2.0 and 4.0 mm), whereas Figure 9b shows equivalent results for different moduli of elasticity (*E_f_* = 150, 200 and 250 GPa). The results in these figures indicate that, as *E_f_* increases, the overall stiffness of the slab also increases, and less deflection is recorded (Figure 9b). When the stress in the CFRP is compared, a thicker CFRP develops less stress than the thinner plate. Therefore, it can be concluded that increasing *E_f_* gives the same results as increasing *t_f_*. However, the results show that the load at which diagonal crack develops changes marginally if *E_f_* changes. The results of the parametric study show that increasing the thickness or the elastic modulus of the FRP plate can reduce the efficiency of the FRP plate bonding technique and make it susceptible to earlier debonding failure or to allow multiple interfacial debonding failure. Therefore, it is proposed to consider end tapering to investigate the increase in effectiveness of the CFRP sheets. The CFRP strengthening is designed according to the bending moment diagram in order to achieve the effective use of the material by tapering the thickness of both sides (see tapering detail in Figure 3a) at the loading point of both ends of the longitudinal CFRP plates.

Table 3 shows the normal stress of CFRP sheets at the concrete–plate interface and tensile failure load for different CFRP thickness. It can be seen that the normal stress in the case of 0.7 to 1.4 mm thickness plate increases by 31% from the case of a 1.4 mm plate. For the case of 2.0 to 4.0 mm thickness, a 37% increase is obtained when compared with the case of a 4.0 mm CFRP. It reveals that end-tapering technique has an advantage in making the FRP plate more effective by reducing the thickness of the FRP plate at the plate end. Another advantage of tapering is that in strengthened slabs without tapering, the end plate or sheets are found to have a high concentration of principal stress and shear stress. Indeed, the termination of a plate or sheet with constant thickness creates an abrupt change in the element stiffness, which creates both shear stress and normal stress concentrations near the end plate or sheet.

## 6. Performance of End Effect for EBR FRP-Strengthened Slabs

**Figure 10:**
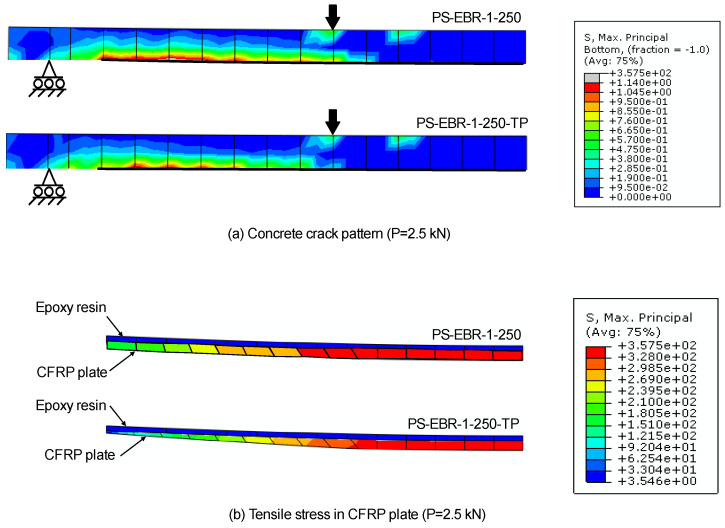
Comparison of principal tensile stress at load P = 2.5 kN of slabs without (PS-EBR-250) and with tapering (PS-EBR-250-TP), (**a**) stress along CFRP sheets and (**b**) detail of tensile stresses at end anchorage of CFRP sheets.

Figure 10a,b show, correspondingly, the contour plots of principal tensile stress (S_max_) and shear stress (S_11_) of the concrete–adhesive interface of the CFRP-strengthened slabs without end-tapering (PS-EBR-250) and with end-tapering (PS-EBR-250-TP). In these plots, the thickness of the CFRP varied from 1.0 to 1.4 mm in PS-EBR-250-TP to compare with the 1.4 mm of CFRP in PS-EBR-250. The development of the principal stresses in the FE model can be used to identify the concentration of stresses in the concrete, CFRP sheets and adhesive resin. As a failure ratio of 0.037 was used (i.e., the tensile stress of concrete input in Abaqus^®^ was 1.14 MPa), a diagonal crack in the concrete would occur when the tensile stress in the concrete (at the end of the plate) reaches its ultimate strength. However, the full composite action is assumed in this modeling exercise, which makes the analysis run completely until a compressive failure of concrete occurs. At this point, the maximum tensile stress in the CFRP sheets can be used to examine the effectiveness of the FRP strengthening before the beam starts to develop diagonal cracking, loses its composite action and eventually fails due to FRP debonding (i.e., Figure 10a). The results confirm that the use of end-tapering at the end anchorage of the CFRP sheets reduces the stresses compared to the slab without end tapering (see Figure 10b).

**Figure 11:**
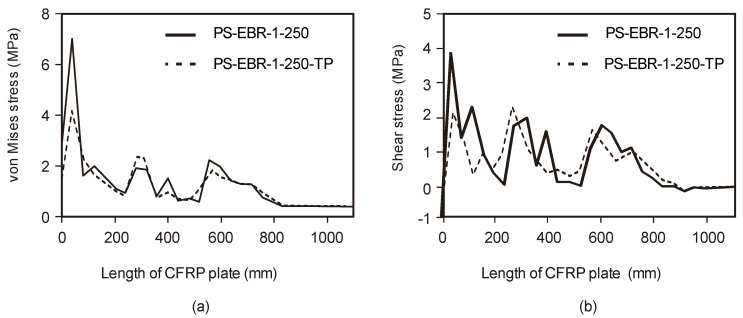
CFRP-concrete interfacial stress (**a**) principal stress and (**b**) shear stress.

Figure 11a compares the CFRP–concrete interfacial principal stresses of slabs without (PS-EBR-250) and with tapering (PS-EBR-250-TP) as a function of the length of the CFRP plate sheets. Likewise, Figure 11a compares analogous results but for shear stresses of such slabs. The results in these figures show that the end-tapering technique has an advantage in making the FRP plate by reducing both von Mises and shear stresses and make it susceptible to early debonding. In these plots, two FRP plates with different thicknesses (constant *t_f_* = 1.4 mm and tapering *t_f_* = 1–1.4 mm) are used to investigate the principal and shear stresses of the slabs and the FRP plates. Figure 11a,b indicate that the principal stress and shear stress concentrate at the end of the CFRP sheets if a constant thickness (*t_f_* = 1.4 mm) is considered. The concentration of these stresses is found to be the cause of debonding due to rip-off or peel-off. Conversely, the tapering reduces the principal stresses (by up to 15%) and shear stresses (by up to 10%) along the anchorage length, thus, re-distributing stresses towards the beam mid-span. Therefore, tapering is extremely successful in reducing the stresses by up to 15%, which can be sufficient to prevent concrete rip-off and peel-off FRP debonding failures.

## 7. Summary and Conclusions

This article numerically examines the behavior of prestressed reinforced concrete slabs strengthened with EBR CFRP sheets in flexure. Computer simulations based on finite element (FE) analysis were conducted on five specimens tested previously by the authors to investigate stress concentrations in the slabs, as well as at the CFRP–concrete bonded interface. The simulation is then used to carry out a parametric study that shows that increasing the thickness or the elastic modulus of the FRP plate affects the efficiency of the FRP plate bonding. Further analysis investigated the efficiency of tapering the EBR FRP on reducing the stress concentrations at the end anchorage to the strengthening system. Based on the results of this study, the following conclusions can be drawn:

- In all CFRP-strengthened prestressed slabs, the ultimate failure was controlled by debonding of the CFRP sheets at the end anchorage. End debonding failure and intermediate crack-induced interfacial debonding failure were observed in the slabs without and with tapered CFRP sheets, respectively.
- The results from the tests showed that an end tapering technique improved the ultimate load by up to 5% compared to slabs without tapering. The tapering also prevented end debonding failure by allowing multiple interfacial debonding failures.
- For CFRP-strengthened slabs, the FE models predicted better the results compared to unstrengthened specimens up to a load level of approximately 75% of the ultimate load. However, the numerical predictions showed approximately 20–25% fewer deflections at ultimate loads. Further research is necessary to achieve better predictions at ultimate loads.
- Results from a parametric study showed that increasing the thickness or the elastic modulus of the CFRP strengthening sheets can affect the efficiency of the FRP plate bonding technique, thus, making it susceptible to potential premature debonding failures.
- The FE results confirmed that a tapering technique at the end anchorage of the CFRP sheets can increase the capacity of a CFRP strengthening system. For the CFRP-strengthened slabs analyzed in this study, the end tapering reduced the stresses by up to 15%, which can be sufficient to prevent concrete rip-off and peel-off debonding failures. Nonetheless, further numerical analyses are necessary to extend the validity of these observations to other structural elements strengthened with EBR CFRP sheets.

## Figures and Tables

**Table 1:** Properties of material used in FE modeling of tested slabs.

Parameters	Tensile Steel	Compressive Steel	CFRP Sheets	Resin
Modulus of elasticity [GPa]	200	206	200	5
Poisson’s ratio	0.29	0.29	0.29	0.35
Yield stress [MPa]	470	360	-	-
Ultimate strength [MPa]	560	400	2590	20
Ultimate plastic strain	0.023	0.0082	-	-

**Table 2:** Concrete parameters adopted in numerical analysis.

Initial elastic modulus	$E_{0}$	22,750	MPa
Poisson’s ratio	𝜈	0.15	-
Compressive cylinder strength	$f_{ck}$	30.8	MPa
Tensile strength	$\sigma_{cr}$	0.037	MPa
Tension stiffening(pre-load)	$\varepsilon_{max}$	0.0007	-
Tension stiffening	$\varepsilon_{max}$	0.0031	-

**Table 3:** Normal stress of CFRP sheets at concrete and tensile failure load for different CFRP thickness.

FRP Thickness *t_f_* (mm)	Normal Stress in FRP $S_{11}$ (MPa)	Load (kN)
0.7	413	4.72
1.4	281	4.35
2.0	254	4.30
4.0	160	4.25

## Data Availability

Not applicable.
